# Ancient Breathing Exercises

**Published:** 1891-03

**Authors:** 


### ANCIENT BREATHING EXERCISES.

Professor Max Müller has recently published some extracts from the Sanscrit, which describe breathing exercises, consisting of deep breathing, expelling and drawing in the breath at a regular rate, and holding the breath, for the purpose, as was said, of steadying the mind. The person taking this exercise was directed to assume a firm and easy position, and then to carefully regulate the breath,—drawing it in through one nostril, closing the other nostril with the finger, then after retaining it for some time, sending it out through the other nostril. The time occupied in the three parts of the exercise,—inhalation, retention, and exhalation,—was regulated by the repetition of the syllable *om*. It was claimed that this method of breathing gave remarkable clearness to the mind, and prepared the individual for a mental state in which most exceptional powers might be manifested.

